# An Efficient Method for No-Reference Video Quality Assessment

**DOI:** 10.3390/jimaging7030055

**Published:** 2021-03-13

**Authors:** Mirko Agarla, Luigi Celona, Raimondo Schettini

**Affiliations:** Department of Informatics, Systems and Communication, University of Milano-Bicocca, Viale Sarca, 336, 20126 Milano, Italy; m.agarla@campus.unimib.it (M.A.); raimondo.schettini@unimib.it (R.S.)

**Keywords:** no-reference video quality assessment, in-the-wild videos, convolutional neural network, support vector regressor, lightweight method, efficient method

## Abstract

Methods for No-Reference Video Quality Assessment (NR-VQA) of consumer-produced video content are largely investigated due to the spread of databases containing videos affected by natural distortions. In this work, we design an effective and efficient method for NR-VQA. The proposed method exploits a novel sampling module capable of selecting a predetermined number of frames from the whole video sequence on which to base the quality assessment. It encodes both the quality attributes and semantic content of video frames using two lightweight Convolutional Neural Networks (CNNs). Then, it estimates the quality score of the entire video using a Support Vector Regressor (SVR). We compare the proposed method against several relevant state-of-the-art methods using four benchmark databases containing user generated videos (CVD2014, KoNViD-1k, LIVE-Qualcomm, and LIVE-VQC). The results show that the proposed method at a substantially lower computational cost predicts subjective video quality in line with the state of the art methods on individual databases and generalizes better than existing methods in cross-database setup.

## 1. Introduction

User-Generated Content (UGC) produced with smartphones or tablets is very prone to natural capture artifacts, such as out of focus, object motion camera shakiness, under/overexposure, sensor noise, adverse weather, and so on. The automatic estimation of the quality of a UGC as perceived by human observers is fundamental for a wide range of applications. For example, to discriminate professional and amateur video content on user-generated video distribution platforms [1], to choose the best sequence among many sequences for sharing in social media [2], to guide a video enhancement process [3], and to rank/choose user-generated videos [4,5].

Blind/No-Reference Video Quality Assessment (NR-VQA) aims toward the development of methods that estimate a quality prediction in close agreement with human judgment in the absence of the pristine video. NR-VQA in the wild is a challenging task not only for the reason that the pristine videos are not available, but also due to the fact that video content is unknown and affected by mixed real-world distortions, especially some of which are temporally heterogeneous (e.g., temporary auto-focus blurs and exposure adjustments). For this reason, methods designed on synthetically distorted video datasets do not scale on natural distortions and more effort needs to be made to develop datasets and methods suitable for NR-VQA in the wild. Recently, some quality databases with UGC videos affected by natural distortions have been published [6,7,8] and the focus of blind video quality prediction methods has gradually shifted to techniques capable of detecting natural video distortions as well [4,5,9]. Although important steps forward have been made from the point of view of the effectiveness of NR-VQA methods on videos in-the-wild, another important aspect that needs to be investigated concerns the efficiency of the proposed methods, especially if they have to be deployed on embedding devices with limited resources such as smartphones and tablets.

**Figure 1:**
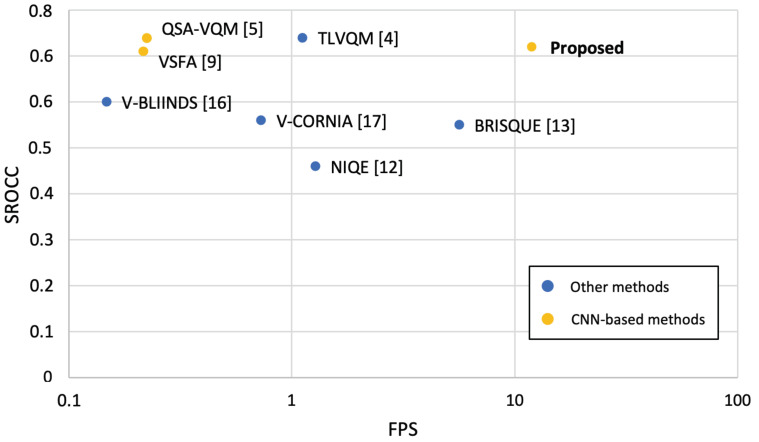
Speed vs. mean Spearman’s Rank-order Correlation Coefficient (SROCC) computed across 100 train-test combinations on LIVE-Qualcomm videos (resolution at 1080p). Speed is measured by running methods in CPU.

In this paper, we mainly focus on the problem of efficiently assessing the quality of in-the-wild videos. The proposed method relies on the insight of our previous article that the combination of quality features with semantic features produces video quality scores in close agreement with human judgment [5]. In this work, we take up the idea of encoding video frames in terms of both semantics and quality and we design a lightweight and efficient method. The proposed method includes a Multi-level feature extraction module as presented in [5]. This consists of two lightweight Convolutional Neural Networks (CNNs) for encoding each video frame in terms of both semantic and quality features. The previous module is followed by a Video quality estimation module that aggregates frame-level features into video-level features by temporal pooling. The spatiotemporal features are finally mapped to a video quality score using a Support Vector Regressor (SVR) machine with Radial Basis Function (RBF) kernel. We design a novel sampling module to select a predefined amount of frames from the entire video sequence. This is motivated by the fact that video signals have temporal redundancy and that the processing of all frames not only represents the bottleneck of our method but also does not significantly improve performance. The proposed method is close in accuracy to benchmark methods but much more efficient, as shown in Figure 1.

The main contributions of this work are the following.

- A lightweight and efficient video quality assessment method for in-the-wild videos using two CNNs thoroughly trained for encoding video frames in terms of both semantics and quality attributes and a Support Vector Regressor (SVR) machine.
- A frame sampling algorithm capable of selecting a predetermined number of frames that exhibit a wide variation in terms of content and/or imaging conditions.
- An evaluation of the proposed method and a comparison with previous VQA methods on four benchmark databases containing UGC videos also in cross-database setup.
- An ablation study that estimates how performance varies as the number of sampled frames changes and that measures the benefits of using our sampling method instead of other strategies.

This paper extends our previous work [5] in three aspects: (1) ResNet-50 [10] architectures exploited in the Multi-level feature extraction module are replaced by the more efficient and lightweight MobileNet-v2 [11], which improves the efficiency without sacrificing performance. (2) Frame-level features are mapped to a video quality score thanks to the Video quality estimation module which is much more simple than the Temporal modeling module. (3) The video quality is estimated by sampling a small set of frames instead of evaluating all frames.

The rest of the paper is organized as follows. Section 2 gives an overview of related works, and the proposed method is detailed in Section 3. Section 4 contains a description of the databases and the training protocol. In Section 5, experimental results are shown and an ablation study where all the investigated methods that allow the definition of the final method are compared. Finally, Section 6 draws conclusions.

## 2. Related Work

Many frame-based NR-VQA methods are based on image quality assessment methods that involve the analysis of Natural Scene Statistics (NSS). Among such methods there are the Naturalness Image Quality Evaluator (NIQE) [12], the Blind/Referenceless Image Spatial Quality Evaluator (BRISQUE) [13], the Feature map-based Referenceless Image QUality Evaluation Engine (FRIQUEE) [14], and the High Dynamic Range Image Gradient-based Evaluator (HIGRADE) [15]. When applied to videos, NSS-based methods measure the deviation of each frame from the natural scene statistics and then average the statistics of all frames to obtain the quality score for the entire video. Few methods in the literature explicitly model temporal features. V-BLIINDS [16] is an extension of the image-based metric that incorporates time-frequency characteristics and temporal motion information. The Video Codebook Representation for No-Reference Image Assessment (V-CORNIA) [17] independently estimates the quality of each video frame thanks to a representation obtained with unsupervised learning and a Support Vector Regression (SVR). Finally, the frame-level quality scores are aggregated through time pooling to obtain the final video quality.

Given the growing interest in the quality assessment of in-the-wild videos, four relevant datasets have been collected and annotated: CVD2014 [18], KoNViD-1k [6], LIVE-Qualcomm [8], and LIVE-VQC [7]. These databases are very challenging, and previous VQA methods, validated on synthetically distorted video datasets, do not produce quality estimates that correlate well with ground-truth Mean Opinion Scores (MOSs). For this reason, methods have been proposed that can capture both spatial and temporal distortions [19,20], some of which by exploiting deep learning-based techniques [5,9,21].

In [19], the natural spatiotemporal scene statistics of natural videos are studied in the spatial domain using 3D-MSCN coefficients and in the frequency domain using spatiotemporal Gabor filters. The previous statistics are then modeled using an Asymmetric Generalized Gaussian Distribution (AGGD). Finally, the AGGD parameters serve as image features and are mapped to a quality score using a SVR. The ChipQA [20] captures both spatial and temporal distortions, by building a representation of local spatiotemporal data that is attuned to local orientations of motion but is studied over large spatial fields. The quality of a video is estimated by identifying and quantifying deviations from the expected statistics of natural, undistorted space-time chips.

SACONVA [22] exploits a 3D shearlet transform for extracting frame-level features, which are then passed to a 1D Convolutional Neural Network (CNN) to predict spatio-temporal quality features. The COnvolutional neural network and Multi-regression-based Evaluation (COME) [23] splits the problem of extracting spatio-temporal quality features into two parts. Spatial quality features of each frame are obtained by computing the max and standard deviation of the activations of the last layer of an AlexNet pretrained for image quality assessment on the CSIQ dataset [24]. Temporal quality features are then extracted as standard deviation of motion scores in the video. Finally, two types of SVR are used in conjunction with a Bayes classifier to predict the video quality score. VSFA [9] integrates into a DNN two eminent effects of human visual system: content dependency and temporal memory effects. It involves the use of a CNN pretrained on Imagenet [25] for encoding video frames, then a Gated Recurrent Unit (GRU) [26] is used for modeling long-term dependencies and predicting frame quality. Finally, a subjectively inspired temporal pooling model provides the overall video quality taking into account the effects of temporal hysteresis. VSFA demonstrated to be very effective on three benchmark video databases: KoNViD-1k, CVD2014, and LIVE-Qualcomm. The Two-Level Video Quality Model (TVLQM) [4] consists of a two-level feature extraction mechanism in which low complexity features are first computed for the full sequence, and high complexity features are then extracted from a subset of representative video frames. The authors further improve their method by combining hand-crafted statistical temporal features from TLVQM and spatial features extracted using 2D-CNN model trained for image quality prediction [21]. In our previous work, we propose the QSA-VQM [5] that exploits two ResNet-50 for encoding a frame at a time in terms of both semantic and quality features, the Temporal modeling block is then in charge of estimating the overall quality score for the video by combining frame features thanks to a Recurrent Neural Network (RNN) and a Temporal Hysteresis Pooling [9]. The Recurrent-In-Recurrent Network (RIRNet) [27] includes two parts: quality degradation learning and motion effect modeling. The first part consists of a ResNet-50 that extracts distortion-aware features from individual frames. The second part comprises a hierarchical temporal model based on RNNs to perform temporal downsampling and aggregation of motion information with different temporal frequencies. Recently, Li et al. [28] propose a unified NR-VQA framework with a mixed datasets training strategy for in-the-wild videos that consists of the previously proposed VSFA as the backbone. The training of the backbone on mixed data is then addressed with two losses, namely, the monotonicity-induced loss and the linearity-induced loss.

## 3. The Proposed Method

**Figure 2:**
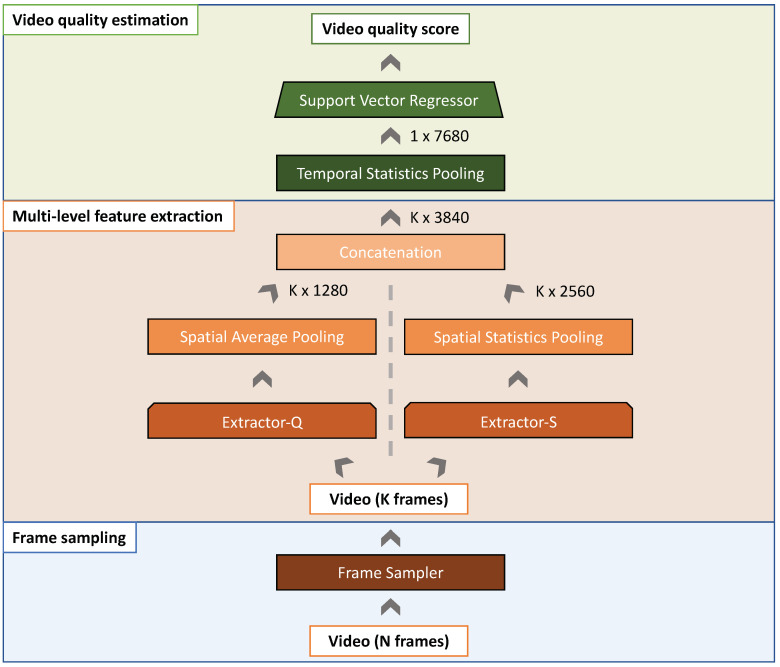
Our NR-VQA method consists of three main steps: the Frame sampling, the Multi-level feature extraction, and the Video quality estimation. In the Frame sampling, a set of *K* frames is first sampled from the whole video. These *K* frames are then encoded in terms of both quality and semantic features in the Multi-level feature extraction step. The Video quality estimation block aggregates the frame-level feature vectors into a video-level feature vector using a Temporal statistic pooling and then estimates the quality score using a Support Vector Regressor.

The proposed method for No-Reference Video Quality Assessment (NR-VQA) follows the insights of our previous QSA-VQM [5], but implements them by adopting design choices aimed at making the method lightweight and efficient enough to be deployed on resource-limited devices. As illustrated in Figure 2, the proposed method estimates the quality score of RGB video sequences of variable resolution and length. It consists of three main modules: the Frame sampling module, the Multi-level feature extraction module, and the Video quality estimation module. In the Frame sampling module, a subset of representative frames is sampled from the entire video. In the Multi-level feature extraction module, the sampled video frames are fed one at a time into two Convolutional Neural Networks (CNNs), called Extractor-Q and Extractor-S, which aim to compute quality and semantic features for each video frame. These features are concatenated and then processed by the Video quality estimation block which aggregates temporal features and exploits a Support Vector Regressor (SVR) machine for predicting a quality score. In the next sections, we detail each block of the proposed method.

### 3.1. Frame Sampling

One of the bottlenecks in video quality assessment is the encoding of all video frames, especially if they are many and have a high resolution. Moreover, we know that temporal redundancy is present in video signals when there is a significant similarity between successive video frames. For both reasons, we propose a sampling algorithm able to maintain video frames that present variations in content and especially in terms of imaging conditions. The HSV color space, which contains the three components hue, saturation, and value, provides an intuitive color representation and is more suitable than the RGB color space to capture features correlated well with human perception [29]. It was also seen that the value component is strongly affected by blur, while the other components remained approximately unchanged. This is due to the high separation between the chromatic and achromatic components in this color space [30]. Thus, in our sampling algorithm we measure the error between frames in the HSV color space.

In Algorithm 1, a subset of *n* frames is sampled from the entire video. Video frames are first rescaled using bilinear interpolation to a resolution with the smaller edge equal to *s* and the other edge adapted to preserve the frame aspect ratio. They are then converted from the RGB to the HSV color space. For each video frame, the Mean Absolute Error (MAE) is calculated with all subsequent frames. Algorithm 2 is applied for selecting the indices of frames having an error higher than a threshold. In order to obtain the required number of frames, *n*, the threshold is optimized using a naive algorithm. The initial threshold is the average of the errors across all video frames, and it is then updated to collect the desired number of frames. To ensure optimal convergence, the delta update is multiplied by a gamma decay factor. In any case, the optimization process stops after a maximum number of iterations, max_iter. In Algorithm 2, we iteratively jump from one frame to the next with an error above the threshold and a minimum number of intermediate frames corresponding to the frame rate *r*. The first condition allows selecting frames with a high difference in terms of content and imaging variations, while the second condition prevents the selection of frames that are too close to the detriment of those that are far away. This condition can occur, for example, in case of camera shake.
**Algorithm 1***sampleFrames(F, n, s, r, max_iter, delta, gamma)*
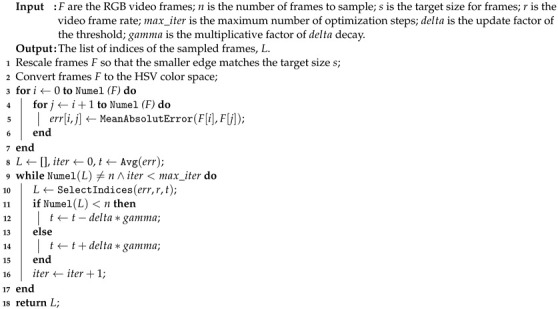

**Algorithm 2***selectIndices(err, r, threshold)*
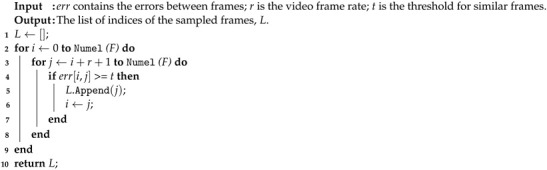



### 3.2. Multi-Level Feature Extraction

Given that human judgments of visual video quality are strongly influenced by the different sensitivity to low-level visual features [31,32] and the semantic video content [33,34], in this work we characterize video frames in terms of these two aspects. To this end, we employ two CNNs that we have called Extractor-Q and Extractor-S to extract low-level quality and semantic features, respectively.

**Figure 3:**
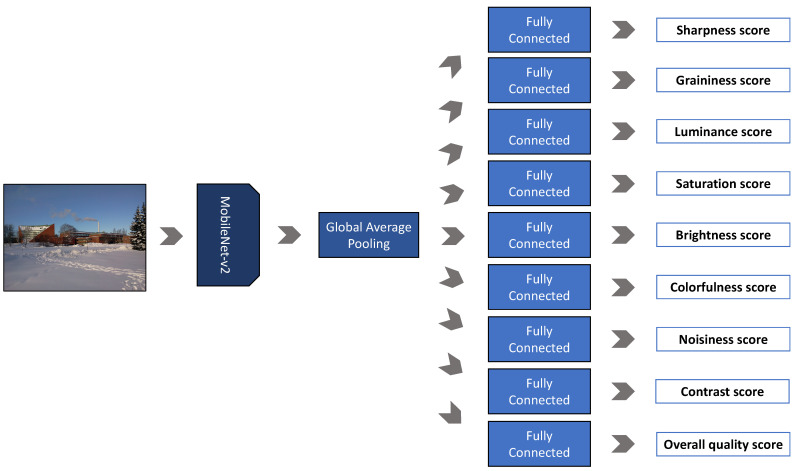
Definition of the Extractor-Q. Given an image of any size, Extractor-Q estimates the overall quality score as well as the scores for eight quality attributes, namely, brightness, colorfulness, contrast, graininess, luminance, noisiness, and sharpness, and color saturation. It consists of a MobileNet-v2 followed by a Global Average Pooling (GAP) level and a fully connected (FC) level for each one of the previously mentioned aspects.

In Figure 3, we show the architecture of the Extractor-Q. It consists of a MobileNet-v2 [11] architecture (given its efficiency and reduced number of parameters [35]) truncated to the last convolutional layer, followed by a Global Average Pooling (GAP) layer. A GAP layer reduces a tensor with dimensions h×w×d in order to have dimensions 1×1×d by simply taking the average of all hw values. Finally, nine different Fully Connected (FC) layers output the scores for the overall image quality and eight image quality attributes, i.e., sharpness, graininess, lightness, color saturation, brightness, colorfulness, contrast, and noisiness. Extractor-Q is trained end-to-end in a multi-task fashion for simultaneously estimating the aforementioned aspects.

To train the Extractor-Q, we combine two databases for image quality assessment: the CID2013 database [36] and the Smartphone Photography Attribute and Quality (SPAQ) database [37]. The first consists of 480 images with resolution 1600×1200 captured by 79 different cameras of varying quality. Each image is annotated by human subjects in terms of overall quality and four attribute scales (i.e., sharpness, graininess, lightness, and color saturation). The SPAQ database contains 11,125 high-resolution pictures taken by 66 smartphones, where each image is annotated in terms of image quality, and image attributes (brightness, colorfulness, contrast, noisiness, and sharpness).

The semantic features for each frame are simply obtained using the Extractor-S which consists in a MobileNet-v2 pretrained on ImageNet for image categorization.

As graphically described in Figure 2, the feature vector for each of the *K* video frames from both CNNs is obtained by truncating the networks to the last convolutional block, which generates an activation volume of m×n×1280, where m×n is the spatial resolution and 1280 is the depth of the volume, respectively. A Spatial Average Pooling (SAP) is applied on the activation volume of the Extractor-Q to reduce the spatial resolution. The resulting feature vector has a shape of K×1280. Instead, a Spatial Statistics Pooling (SSP) [38] is applied by calculating and concatenating the mean and standard deviation of spatial features of the activation volume produced by the Extractor-S. The feature vector obtained by a SSP has a shape K×2560. The output of the Multi-level feature extraction block is obtained by concatenating the feature vectors of each network. A video is represented by a feature vector of K×3840.

### 3.3. Video Quality Estimation

We exploit a Temporal Statistics Pooling to aggregate spatial features obtained from the Multi-level feature extraction into a spatiotemporal feature vector of length equal to 7680. In practice, we found that the use of mean and standard deviation of the features obtained for the various frames of the video does not sacrifice much the performance. The use of a parameterless layer makes our Video quality estimation module very efficient and lightweight. The video level spatiotemporal features are mapped into video quality scores using a Support Vector Regressor (SVR) machine. Specifically, we exploit a SVR with a Radial Basis Function (RBF) kernel as it has shown better performance than simple linear regression and SVR with other kernels.

### 3.4. Implementation Details

For frame sampling, we set the frame target size *s* to 16, the value for *r* is equal to half the frame rate value, the number of frames to sample *n* is 15, max_iter for the optimization process is equal to 20, the update factor delta is 0.005, and gamma corresponds to 0.25.

The training of our NR-VQA method takes place in two stages. In the first stage, the two CNNs of the Multi-level feature extraction block are used to encode the frames are trained. These two training processes are conducted using the PyTorch framework [39]. In the second stage, the SVR of the Video quality estimation block is trained. We use the SVR provided by the Scikit-learn library [40]. For the Extractor-S we use the ImageNet pretrained MobileNet-v2 provided by Torchvision package of the PyTorch framework [39]. Training images are randomly cropped to 224×224 pixels and horizontally flipped. As mentioned in Section 3.2 the Extractor-Q is trained on the combination of two datasets (i.e., CID2013 and SPAQ) for the estimation of the overall quality and quality attributes. Furthermore, in this case we start from a MobileNet-v2 pretrained on Imagenet, and we use the initialization technique proposed in [41] for the fully connected layers predicting the scores for each attribute. Image labels for each quality attribute are mapped in range [0, 1] using the min-max scaling. Adam is chosen as optimizer, while the linear combination of a Mean Absolute Error (MAE) loss for each task is used as optimization criterion. We train the network with a fixed learning rate equal to $1\times 10^{-4}$ for 150 epochs on the entire dataset with the batch size equal to 4. To allow the network to be less sensitive to changes in resolution, we propose a multi-scale training procedure in which a crop is extracted for each image of CID2013 and SPAQ by randomly sampling the position and choosing one crop size randomly from the following: 854×480 (480p), 1280×720 (720p), and 1920×1080 (1080p). The size of the crop is adapted if the training image is not large enough. The horizontal flip is then applied randomly to increase the data.

The SVR with RBF kernel has two hyperparameters that need to be tuned: the kernel parameter γ for the RBF kernel and the soft-margin parameter *C* trading off complexity and data misfit. We select these hyperparameters by running a Bayesian optimization framework [42]. The latter uses a surrogate model to approximate the objective function and chooses to optimize it according to some acquisition function. The surrogate model used is Random Forest, while the acquisition function is Upper Confidence Bound (UCB). The search value ranges for *C* and γ are [0.01,5] and $\left[1\times 10^{-4},0.1\right]$, respectively.

The training set data are split into 80% train and 20% validation. The SVR is trained with a pair of hyperparameters—*C* and γ, and then the Spearman’s Rank-order Correlation Coefficient (SROCC) is calculated on the validation data. The above procedure is repeated with the same pair of hyperparameters for 100 times (generating new train–val splits) and the average of the SROCCs obtained over all times is calculated as the evaluation metric of the hyperparameters pair. The pair of hyperparameters that produces the highest SROCC mean is the one chosen. The optimization consists of 600 iterations. We use SROCC instead of PLCC to avoid overfitting on validation data. As PLCC evaluates the goodness of the linear relationship between the MOS and the predicted scores, it may find hyperparameters that perform well on the validation data but do not generalize on the test data. Instead, SROCC relax the linearity constraint and helps find the hyperparameters that just ensure monotonicity between MOS and predicted scores.

## 4. Experiments

In this section, we first describe the databases considered for the experiments, we then present the experimental setup and the evaluation criteria.

### 4.1. Databases for In-the-Wild Video Quality Assessment

There are four publicly available databases for video quality assessment in-the-wild: Camera Video Database (CVD2014) [18], Konstanz Natural Video Database (KoNViD-1k) [6], LIVE-Qualcomm Mobile In-Capture Video Quality Database (LIVE-Qualcomm) [8], and LIVE Video Quality Challenge Database (LIVE-VQC) [7].

CVD2014 [18] is a collection of 234 videos of resolution 640×480 or 1280×720 recorded by 78 different cameras (from low-quality mobile phone cameras to high-quality digital single lens reflex cameras). Each video captures one among five different scenes and presents distortions related to the video acquisition process. The trimmed videos have lengths of 10–25 s with 11–31 fps. The realignment Mean Opinion Scores (MOSs) lay in the range [−6.50, 93.38].

The KoNViD-1k database [6] contains 1200 videos of resolution 960×540 sampled according to six specific attributes from the YFCC100M dataset [43]. The resulting database contains video sequences of 8 s with a wide variety of contents and authentic distortions. The MOS have been collected through a crowdsourcing experiment and ranges from 1.22 to 4.64.

The LIVE-Qualcomm database [8] consists of 208 videos of resolution 1920×1080 captured by eight different smartphones. These videos have a length of 15 s and are affected by six in-capture distortions: artifacts, color, exposure, focus, sharpness, and stabilization. A subjective study has been conducted under two different study protocols in a controlled laboratory. A total of 39 subjects has been randomly assigned to one of the setups. In this work, we consider the unbiased MOS scores gathered while the subject freely watch videos. The obtained MOS belong to the range [16.56, 73.64].

Finally, the LIVE Video Quality Challenge (LIVE-VQC) database [7] contains 585 videos of unique content, captured by 101 different devices (the majority of these were smartphones), with a wide range of complex authentic distortions. Videos are on average 10 s long and have variable resolutions, but most videos have resolution equal to 404×720, 1024×720, and 1920×1080. Subjective video quality scores have been collected via crowdsourcing: a total of 4776 unique participants produced more than 205,000 opinion scores. MOS span between 0 and 100.

**Figure 4:**
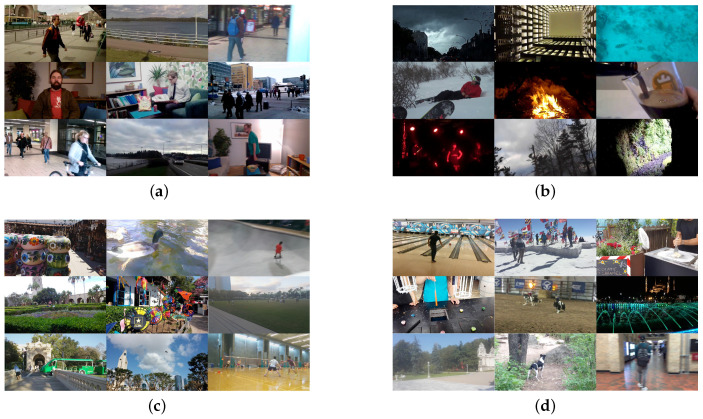
Sample frames of the video contents contained in the four considered databases: (**a**) CVD2014, (**b**) KoNViD-1k, (**c**) LIVE-Qualcomm, and (**d**) LIVE-VQC.

An overview of database properties is provided in Table 1, while frames samples are in Figure 4.

### 4.2. Experimental Setup

The evaluation metrics for NR-VQA methods are Pearson’s Linear Correlation Coefficient (PLCC), Spearman’s Rank-order Correlation Coefficient (SROCC), and Root Mean Square Error (RMSE).

The PLCC measures the linear correlation between the actual and the predicted scores, and it is defined as

(1) $$PLCC=\frac{\sum_{i}^{N}\left(x_{i}-\bar{x}\right)\left(y_{i}-\bar{y}\right)}{\sqrt{\sum_{i}^{N}{\left(x_{i}-\bar{x}\right)}^{2}}\sqrt{\sum_{i}^{N}{\left(y_{i}-\bar{y}\right)}^{2}}},$$

where *N* is the number of samples, $x_{i}$ and $y_{i}$ are the sample points indexed with *i*, and finally $\bar{x}$ and $\bar{y}$ are the means of each sample distribution. Instead, the SROCC estimates the monotonic relationship between the actual and the predicted scores, and it is calculated as

(2) $$SROCC=1-\frac{6\sum_{i}^{N}d_{i}^{2}}{N(N^{2}-1)},$$

*N* is the number of samples, and $d_{i}=\left(\mathrm{rank}\left(x_{i}\right)-\mathrm{rank}\left(y_{i}\right)\right)$ is the difference between the two ranks of each sample.

Finally, the RMSE measures score accuracy and it is defined as

(3) $$RMSE=\sqrt{\frac{1}{N}\sum_{i}^{N}{\left(x_{i}-y_{i}\right)}^{2}},$$

where *N* is again the number of samples, while $x_{i}$ and $y_{i}$ are the sample points indexed with *i*.

For the experiments, the same experimental protocol used in [4,8] was followed. It consists in running 100 times the random selection of 80% of training videos and 20% testing videos. Precisely, we exploit the same 100 splits used in [9] that do not prevent the same scene to be both in training and evaluation sets. This fact can cause a bias in the resulting performance especially for the CVD2014, which only has five different scenes. However, applying another experimental protocol would have introduced other problems such as the imbalanced of sample number between the train split and the test split. For the sake of coherence, we train and measure the performance of other methods on the same splits.

## 5. Results

In this section, we compare the performance achieved by our method with those obtained by the previous NR-VQA methods for each of the considered databases. Furthermore, we conduct a performance evaluation of the generalization ability of the proposed method in cross-database scenarios, which are more challenging due to different types of contents and degradation characteristics. Finally, we report an ablation study in which the different choices that have been investigated during the design of the proposed method are compared.

### 5.1. Performance on Single Databases

The experimental results are reported in terms of average PLCC, SROCC, and RMSE across the 100 iterations of train–test random splits for all the considered databases (CVD2014, KoNViD-1k, LIVE-Qualcomm, and LIVE-VQC). We compare the proposed method with several benchmark methods, namely, NIQE [12], BRISQUE [13], V-CORNIA [44], V-BLIINDS [16], HIGRADE [15], TLVQM [4], VSFA [9], and QSA-VQM [5]. For the sake of comparison, the same random train–test splits were used for all the methods (For reproducible research, we make the 100 train–test splits available at https://rb.gy/tyhlk1; accessed on 13 March 2021). Table 2 shows the average of the considered metrics and their corresponding standard deviations. We can draw several conclusions from these results. First, one can see that TLVQM, VSFA, QSA-VQM, and the proposed method achieve very similar performance on all the considered databases. This is an interesting result considering that the other methods explicitly model time distortions frame-by-frame, while our method aggregates the features of few sampled frames. Second, our method obtains the second-best performance after TLVQM and QSA-VQM with a gap of 0.02 for all metrics. For LIVE-VQC, the proposed method achieves exactly the same PLCC, while the values of SROCC and RMSE are worse than TLVQM. We attain this accurate model but with much lower computational cost, as we will see in Section 5.3.

**Figure 5:**
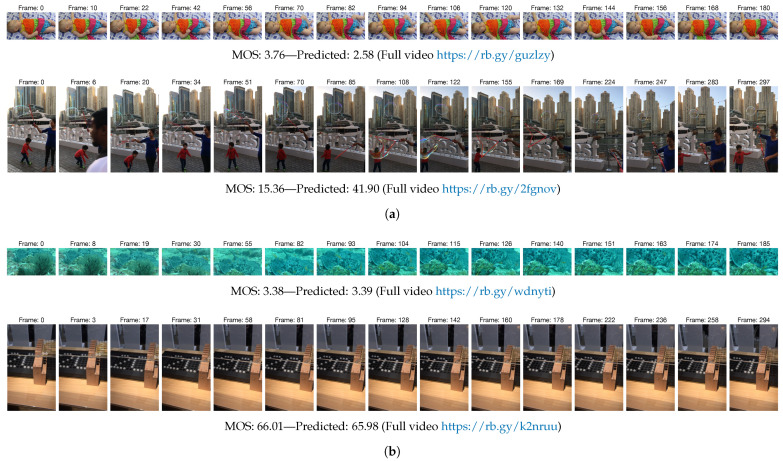
*(Best viewed in colors and magnified.)* Visualization of some examples of prediction of the proposed method. (**a**) Two samples of under- and overestimated video quality predictions. (**b**) Two videos with a very low error between the MOS and the predicted quality score. For each video, the 15 frames sampled by the proposed method, the MOS, and the quality score predicted by our method are shown.

In Figure 5a, we report two video sequences, belonging to KoNViD-1k and LIVE-VQC, where our method over- or underestimates the overall quality. To better understand why the method estimates these quality scores, we provide the sampled frames. The quality score for the KoNViD-1k video sequence has been underestimated by the proposed method. This is probably motivated by the fact that, although there are no motion artifacts present, the video content is slightly out of focus. For LIVE-VQC video, our method predicts a higher quality score than MOS. Looking at the video, no particular quality impairments are evident apart from the beginning of the sequence, probably the low MOS is due to the fact that there is no main subject always visible. However, in our opinion the provided MOS equal to 15.36 does not reflect the objective quality of the video. Figure 5b shows a sample of KoNViD-1k and one of LIVE-VQC for which the proposed method estimates a quality score that almost exactly coincides with the MOS. Both predictions make sense, in fact the first video shows an underwater scene in which the camera rarely moves, while the second is a static shot indoor with the right lighting conditions and excellent quality.

**Figure 6:**
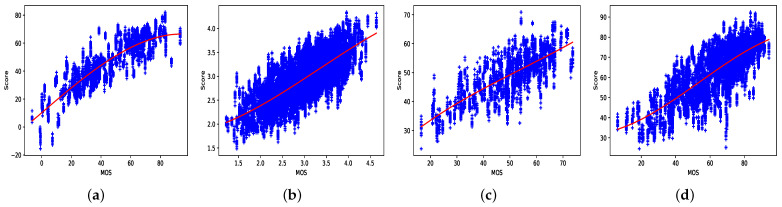
Scatter plots of the predicted scores versus MOS for the four considered databases: (**a**) CVD2014, (**b**) KonViD-1k, (**c**) LIVE-Qualcomm, and (**d**) LIVE-VQC.

Figure 6 shows the scatter plots on the four databases. They report the MOS with respect to the corresponding predicted scores for all the samples considered in the 100 iterations. A logistic regression function is drawn for highlighting the silhouette of the fit. We can observe that apart for LIVE-Qualcomm and LIVE-VQC, the other distributions are well fit.

### 5.2. Performance across Databases

After demonstrating the effectiveness on different databases, in this section we focus on cross-database experiments to verify the robustness and generalization capacity of the proposed method. To this end, for each training database we took the 100 trained models and used them to estimate the quality scores of all the videos from the other databases. Finally, we reported the average and the standard deviation on the 100 iterations for each test database. We compare the performance of the proposed method with state-of-the-art methods which achieved similar performance on the different databases, namely, TLVQM, VSFA, and QSA-VQM. Table 3 reports the comparison of our method with the competitors when it is trained on a database and tested on the remaining three. We emphasize that our method generalizes well when trained on CVD2014, LIVE-Qualcomm, and LIVE-VQC, while performance is low on the other databases when the method is trained on KoNViD-1k. It is possible to see that in general the correlation between LIVE-VQC and KoNViD-1k is higher than other databases. This might be because the video content and the subjective study for collecting human judgments are similar.

### 5.3. Computation Time

For NR-VQA methods, efficiency is also crucial. In this section, we complement the part of performance estimation with that of computational efficiency. We measure the computational efficiency of several methods on the same desktop computer with an Intel Core i7-7700 CPU@3.60GHz, 16 GB DDR4 RAM 2400 MHz, and NVIDIA Titan X Pascal with 3840 CUDA cores. The operating system is Ubuntu 16.04. We compare computation time of our method with the one of BRISQUE, NIQE, TLVQM, V-CORNIA, V-BLIINDS, VSFA, and QSA-VQM. Most of the methods are implemented in MATLAB, TLVQM has the feature extraction part in MATLAB and the regression part in Python 3.6. VSFA, QSA-VQM, and our method are implemented in Python 3.6 and exploit the PyTorch 1.5.1 framework. For estimating the computation time of all methods, we run the original codes using default settings without any modification in CPU. As in [9], we select four test videos with different lengths and different resolutions: 240 frames video with resolution 960×540 pixels, 346 frames at a resolution of 640×480, 467 frames at a resolution of 1280×720, and 450 frames at a resolution of 1920×1080. We repeat the tests ten times and the average computation time (seconds) for each method is shown in Table 4. The proposed method is extremely faster than the others at all resolutions and the gap increases as the video resolution increases. In particular, it is 2× faster than BRISQUE that is the second method in terms of efficiency but much less accurate as we previously show in Table 2. At the bottom of the table, we report results in GPU mode for VSFA, QSA-VQM, and our method: the only three methods exploiting GPU accelerations among the compared methods. These methods in GPU mode can be about 32× faster than the CPU mode. We highlight that the proposed method is 12× faster than VSFA at 540p, which is the second fastest method in GPU and achieves comparable performance to our method.

**Figure 7:**
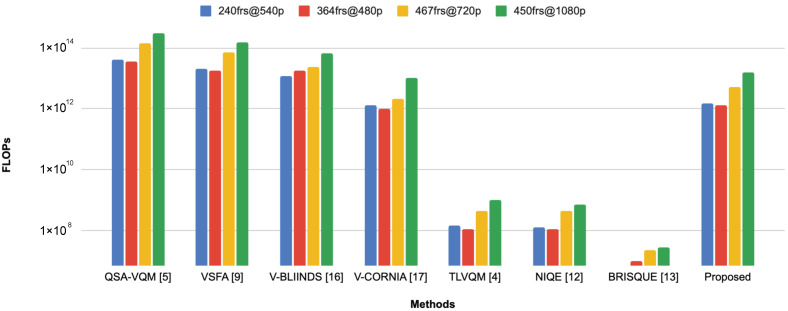
Floating point operations (FLOPs) for each method estimated for videos with different number and resolution of frames.

To complement the previous analysis we also estimate the number of floating point operation (FLOPs) for previous methods and we compare them with the one of our method. Figure 7 shows the FLOPS as a function of number of frames and resolution for the considered methods. Both QSA-VQM [5] and VSFA [9] have a very large number of FLOPs, while BRISQUE [13] is the method with the lowest order of magnitude of FLOPs (about $10^{7}$). The proposed method has an order of magnitude of FLOPs equal to $10^{12}$ which is higher than that of BRISQUE. This gap was presumable but is not reflected in the computation time because, although the operations of the proposed method are much more than those of BRISQUE, they are parallelized. Therefore, it turns out that, compared to BRISQUE, the proposed method has a lower computation time in the CPU and extremely lower in GPU mode.

### 5.4. Ablation Study

In this section, we present the alternative design choices that have been investigated to lead us to the definition of the final model. In particular, we compare several design choices adopted for the frame sampling module and the multi-level feature extraction module, respectively.

**Figure 8:**
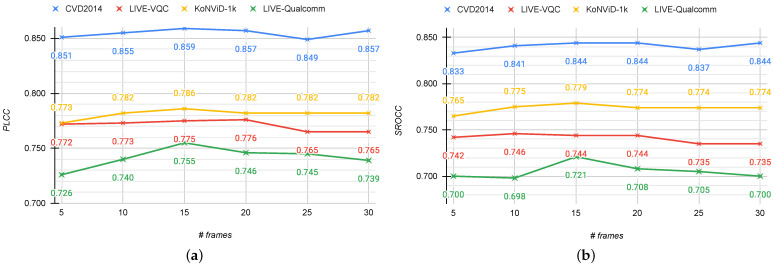
Mean PLCC (**a**) and SROCC (**b**) across 100 train–test random splits of the four considered datasets with respect to the number of sampled frames.

**Frame sampling.** We assess the performance by varying the number of sampled frames, the size of the minimum step (*r*) to sample the frames, and the size of the minimum edge to which to resize the frames (*s*). We also compare the proposed sampling algorithm with other solutions. We perform experiments on all databases with a number of sampled frames ranging from 5 to 30 with a step of 5. Figure 8 shows the plots for PLCC and SROCC with respect to the number of sampled frames for all the considered databases. It is possible to see that the proposed method obtains the best correlations on all databases for a number of frames equal to 15. This is especially noticeable for the LIVE-Qualcomm database for which the performance initially increases, reaches the peak in correspondence of 15 frames, and then slightly decreases.

**Figure 9:**
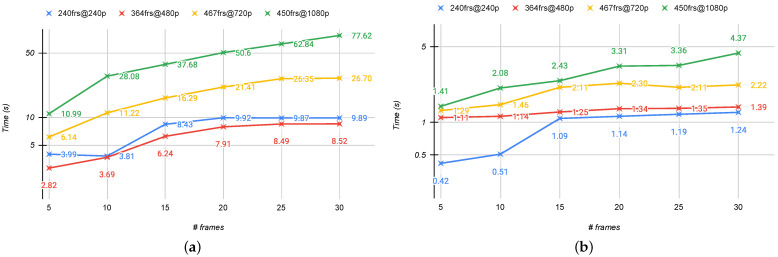
Computation time comparison in seconds for four videos selected from the considered databases with respect to the number of frames sampled. (**a**) The time in CPU; (**b**) the time in GPU. {xxx}frs@{yyy}p indicates the video frame length and the resolution, respectively.

In Figure 9, the graphs showing the computation time with respect to the number of sampled frames are reported. We estimate this metric for four videos with the same characteristics as those used in Section 5.3. As expected, the computation time increases as the resolution and number of frames increase. For example, running the proposed method for NR-VQA with 5 frames instead of 30 results in a 3× increase in compute time in GPU mode. This gap is more noticeable in the CPU rather than GPU mode. To summarize, this analysis confirms that the number of frames to process is a bottleneck for our method.

**Figure 10:**
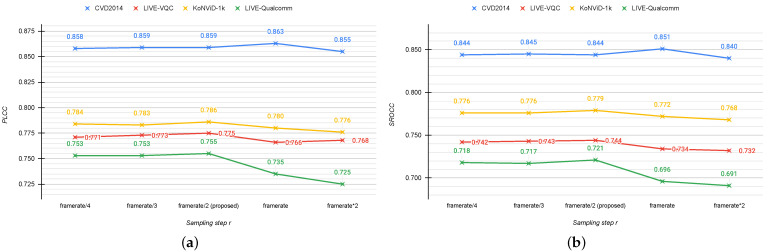
Mean PLCC (**a**) and SROCC (**b**) computed across 100 train–test combinations on the considered databases for different sampling step (r) values in the proposed frame sampling algorithm.

**Figure 11:**
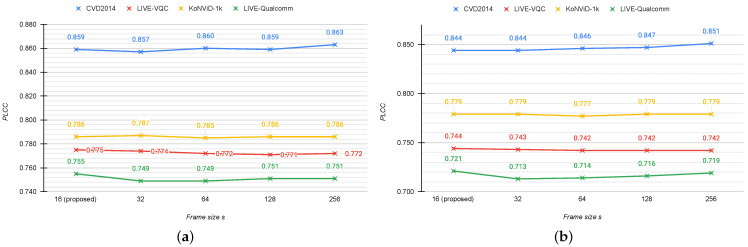
Mean PLCC (**a**) and SROCC (**b**) computed across 100 train–test combinations on the considered databases using different frame size (s) values in the proposed frame sampling algorithm.

We evaluate how the performance varies when modifying the minimum step (*r*) to sample frames in the proposed algorithm. We choose five different values for *r*: framerate/4, framerate/3, framerate/2, framerate, and framerate×2. Figure 10 shows the results in terms of PLCC and SROCC. As can be seen, the best correlation is obtained for *s* equal to framerate/2, while the performance worsens for higher steps. This behavior occurs for all databases except for CVD2014. We also measure the impact of the frame size on the sampling algorithm. Specifically we choose five different sizes to re-scale the short edge of the frames: 16, 32, 64, 128, and 256. These values have been chosen in order to reduce the computation time taking into account the possible masking effect of the artifacts within frames. Figure 11 presents PLCC and SROCC on the four considered databases varying the frame size (*s*) in the frame sampling module. We point out that the performance is not significantly different as the size of the frame increases. This means that even if the size of the frames is very small, it does not impact the choices that our algorithm makes. As there is not a big difference in terms of correlation, we choose s=16 because it results in a huge time gain for the sampling module (about 80 times faster than s=256 on videos at 1080p with 450 frames).

**Figure 12:**
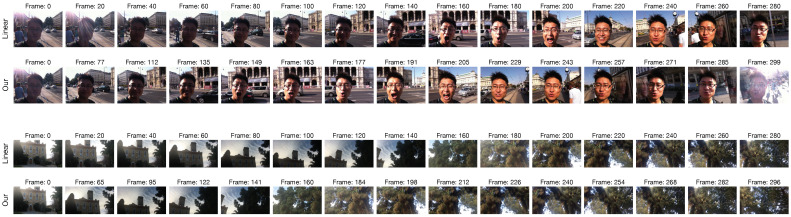
*(Best viewed in colors and magnified.)* Linear vs. our frame sampling. Two example videos are shown in which the 15 frames sampled linearly or with the proposed sampling algorithm are compared.

To better understand the actual benefit provided by the proposed frame sampling algorithm, we compare its performance with those obtained by considering all video frames, by linearly sampling frames from the whole video, or by selecting the frames with the highest MAE. The latter is implemented by simply taking the first *K* frames with the highest MAE with respect to the previous. Table 5 reports the comparison among the three sampling algorithms for the four considered databases. Our sampling algorithm achieves the best PLCC, SROCC, and RMSE on LIVE-Qualcomm and LIVE-VQC databases, while it achieves performances equal to those obtained by taking all frames on CVD2014 and KoNViD-1k. Linear and MAE samplings perform worse than the other two approaches. This is particularly true for the MAE sampling, which obtains the worst performance compared to all variants on all the considered databases apart from LIVE-Qualcomm, where it obtains the second best result. The most important consideration is that using 15 frames instead of all frames makes our method faster by 5× in GPU and 15× in CPU, respectively. In Figure 12, the 15 frames sampled by the linear and the proposed sampling algorithms for two video sequences are compared. As it is possible to see for the examples shown, our algorithm chooses very different frames both for the type of content and for the imaging conditions.

**Multi-level feature extraction.** We evaluate the performance of the proposed NR-VQA method considering the logits, rather than the features, as outputs of Extractor-Q and Extractor-S, respectively. To this end, we follow the same procedure described in Section 3.2 with the only difference that we do not truncate the networks but we use them in their entirety. For the extractor-Q, given a video frame of any size, we get an output equal to m×n×9, where m×n is the spatial resolution and 9 are the values for the quality score and the eight quality attributes. The SAP layer is then applied to reduce the spatial resolution and obtain a feature vector of size 9. The Extractor-S predicts a volume equal to m×n×1000, where m×n is the spatial dimension, while 1000 are the semantic classes of ImageNet. The SSP layer then reduces the spatial resolution, and the resulting feature vector has size 2000. At this point the features are concatenated. A video of *K* frames is finally represented by a feature vector of K×2009. Table 6 reports the results for the four considered databases. We highlight that the performance achieved by our method exploiting the logits instead of the features are lower on all the databases. This may be justified by the fact that the feature vector is more informative than logits where a problem of masking of the various attributes may occur. Furthermore, there is a computational advantage due to the fact that we exclude the fully connected layers.

## 6. Conclusions

We introduced an effective and efficient NR-VQA method for in-the-wild videos. It consists of a sampling algorithm that removes temporal redundancy by selecting a set of representative frames. These frames are passed to two lightweight CNNs that encode both the quality attributes and the semantic content for each frame. Frame-level features are then aggregated into video-level features and finally mapped to a quality score using a SVR. Experiments on four recent large-scale UGC video databases show the accuracy of the proposed method. Cross-database experiments also showed that the proposed method is more robust and generalizes better than the algorithms in the literature. Finally, an analysis of the computational efficiency of methods highlights that the proposed method is several orders of magnitude less expensive than methods achieving very similar accuracy. It runs at a speed up to 185 FPS on one NVIDIA X Pascal GPU and 12 FPS on one Intel i7-770 CPU for 1080p videos. At the same resolution, TLVQM and QSA-VQM, when achieving the same accuracy, are approximately 10× and 50× slower than the proposed method in CPU (see Figure 1).

The sampling algorithm proposed in this article could bring benefits to state-of-the-art NR-VQA methods. In the future, we intend to conduct a comprehensive analysis to assess what are the pros and cons of its adoption.

## Figures and Tables

**Table 1:** Overview of the publicly available databases for in-the-wild video quality assessment. In the column *Device types*: “DSLR” stands for Digital single lens reflex.

Attribute/Database	CVD2014 [18]	KoNViD-1k [6]	LIVE-Qualcomm [8]	LIVE-VQC [7]
Year	2014	2017	2017	2018
No. of sequence	234	1200	208	585
No. of scenes	5	1200	54	585
No. of devices	78	N/A	8	101
Device types	smartphone and DSLR	DSLR	smartphone	smartphone
Distortion type	generic	generic	specific	generic
Duration	10–25 s	8 s	15 s	10 s
Resolution	VGA and 720 p	540 p	1080 p	various
Frame rate	10–31	30	30	N/A
Format	various	MPEG-4	YUV	N/A
Rating per video	27–33	50	39	>200
MOS range	–6.50–93.38	1.22–4.64	16.56–73.64	0–100

**Table 2:** Mean Pearson’s Linear Correlation Coefficient (PLCC), Spearman’s Rank-order Correlation Coefficient (SROCC), and Root Mean Square Error (RMSE) across 100 train–test combinations on the four considered databases. In each column, the best and second-best values are marked in **boldface** and underlined, respectively.

	CVD2014			KonViD-1k		
	PLCC ↑	SROCC ↑	RMSE ↓	PLCC ↑	SROCC ↑	RMSE ↓
NIQE [12]	0.61±0.09	0.58±0.10	17.10±1.5	0.34±0.05	0.34±0.05	0.61±0.03
BRISQUE [13]	0.67±0.09	0.65±0.10	15.90±1.8	0.58±0.04	0.56±0.05	0.52±0.02
V-CORNIA [17]	0.71±0.08	0.68±0.09	15.20±1.6	0.51±0.04	0.51±0.04	0.56±0.02
V-BLIINDS [16]	0.74±0.07	0.73±0.08	14.60±1.6	0.64±0.04	0.65±0.04	0.49±0.02
HIGRADE [15]	0.76±0.06	0.74±0.06	14.20±1.5	0.72±0.03	0.73±0.03	0.44±0.02
TLVQM [4]	0.80±0.04	0.80±0.04	12.89±1.2	0.76±0.02	0.76±0.02	0.42±0.02
VSFA [9]	0.86±0.03	0.86±0.05	11.35±1.4	0.79±0.02	0.78±0.03	0.41±0.03
QSA-VQM [5]	0.87±0.04	0.86±0.04	11.03±1.4	0.81±0.02	0.81±0.02	0.39±0.03
Proposed	0.86±0.04	0.84±0.04	$\underline{11.13\pm 1.24}$	0.79±0.02	0.78±0.02	0.40±0.02
	**LIVE-Qualcomm**			**LIVE-VQC**		
	**PLCC**↑	**SROCC**↑	**RMSE**↓	**PLCC**↑	**SROCC**↑	**RMSE**↓
NIQE [12]	0.48±0.12	0.46±0.13	10.70±1.3	0.58±0.05	0.56±0.06	13.86±0.7
BRISQUE [13]	0.54±0.10	0.55±0.10	10.30±0.9	0.64±0.06	0.59±0.07	13.10±0.8
V-CORNIA [17]	0.61±0.09	0.56±0.09	9.70±0.9	0.72±0.04	0.67±0.05	11.83±0.7
V-BLIINDS [16]	0.67±0.09	0.60±0.10	9.20±1.0	0.72±0.05	0.69±0.05	11.76±0.8
HIGRADE [15]	0.71±0.08	0.68±0.08	8.60±1.1	0.63±0.06	0.61±0.07	13.03±0.9
TLVQM [4]	0.77±0.06	0.74±0.07	7.62±1.0	0.78±0.04	0.78±0.04	10.75±0.9
VSFA [9]	0.75±0.09	0.71±0.10	8.31±1.1	0.75±0.04	0.69±0.05	11.72±0.9
QSA-VQM [5]	0.77±0.06	0.74±0.07	7.93±1.0	0.78±0.04	0.74±0.05	11.06±0.8
Proposed	0.76±0.07	0.72±0.08	8.04±0.80	0.78±0.04	0.74±0.04	10.85±0.6

**Table 3:** SROCC in the Cross-dataset setup. In each column, the best and second-best values are marked in **boldface** and underlined, respectively.

Training	CVD2014			KoNViD-1k		
Testing	LIVE-Qualcomm	KoNViD-1k	LIVE-VQC	CVD2014	LIVE-Qualcomm	LIVE-VQC
TLVQM [4]	0.35±0.08	0.29±0.11	0.41±0.09	0.39±0.08	0.41±0.06	0.50±0.06
VSFA [9]	0.34±0.06	0.55±0.04	0.46±0.04	0.65±0.04	0.60±0.04	0.70±0.01
QSA-VQM [5]	0.37±0.06	0.57±0.04	0.41±0.07	0.67±0.04	0.64±0.03	0.66±0.02
Proposed	0.32±0.14	0.63±0.04	0.62±0.06	0.55±0.12	0.58±0.09	0.71±0.05
**Training**	**LIVE-Qualcomm**			**LIVE-VQC**		
**Testing**	**CVD2014**	**KoNViD-1k**	**LIVE-VQC**	**CVD2014**	**LIVE-Qualcomm**	**KoNViD-1k**
TLVQM [4]	0.48±0.07	0.49±0.04	0.53±0.35	0.49±0.04	0.48±0.04	0.56±0.04
VSFA [9]	0.48±0.07	0.64±0.02	0.63±0.02	0.48±0.06	0.56±0.03	0.67±0.02
QSA-VQM [5]	0.53±0.06	0.62±0.02	0.60±0.03	0.47±0.06	0.40±0.07	0.59±0.05
Proposed	0.50±0.14	0.63±0.05	0.69±0.05	0.54±0.12	0.62±0.09	0.68±0.03

**Table 4:** Computation time comparison in seconds for four videos selected from the considered databases. {xxx}frs@{yyy}p indicates the video frame length and the resolution, respectively.

Mode	Method	240frs@540p	364frs@480p	467frs@720p	450frs@1080p
CPU	V-BLIINDS [16]	382.06	361.39	1391.00	3037.30
	QSA-VQM [5]	281.21	265.13	900.72	2012.61
	VSFA [9]	269.84	249.21	936.84	2081.84
	V-CORNIA [17]	225.22	325.57	494.24	616.48
	TLVQM [4]	50.73	46.32	136.89	401.44
	NIQE [12]	45.65	41.97	155.90	351.83
	BRISQUE [13]	12.69	12.34	41.22	79.81
	Proposed	8.43	6.24	16.29	37.68
GPU	QSA-VQM [5]	9.70	9.15	25.79	55.27
	VSFA [9]	8.85	7.55	27.63	58.48
	Proposed	0.69	0.85	1.71	2.43

**Table 5:** Mean PLCC, SROCC, and RMSE across 100 train–test combinations on the four considered databases using different sampling methods. In each column, the best and second-best values are marked in **boldface** and underlined, respectively.

	CVD2014			KoNViD-1k		
	PLCC ↑	SROCC ↑	RMSE ↓	PLCC ↑	SROCC ↑	RMSE ↓
All frames	0.856±0.040	0.845±0.038	11.229±1.215	0.786±0.021	0.779±0.023	0.400±0.017
Linear sampling (15frs)	0.850±0.041	0.838±0.039	11.455±1.234	0.783±0.021	0.775±0.024	0.403±0.016
MAE sampling (15frs)	0.842±0.042	0.831±0.045	11.701±1.242	0.745±0.027	0.741±0.029	0.429±0.019
Proposed sampling (15frs)	0.859±0.036	0.844±0.039	11.129±1.214	0.786±0.021	0.779±0.024	0.401±0.017
	**LIVE-Qualcomm**			**LIVE-VQC**		
	**PLCC**↑	**SROCC**↑	**RMSE**↓	**PLCC**↑	**SROCC**↑	**RMSE**↓
All frames	0.600±0.107	0.581±0.105	10.357±0.917	0.774±0.037	0.741±0.044	10.878±0.553
Linear sampling (15frs)	0.600±0.107	0.583±0.104	10.379±0.919	0.765±0.039	0.730±0.047	11.053±0.542
MAE sampling (15frs)	0.705±0.075	0.676±0.081	8.169±0.891	0.731±0.046	0.703±0.047	11.712±0.627
Proposed sampling (15frs)	0.755±0.071	0.721±0.077	8.037±0.803	0.775±0.037	0.744±0.044	10.854±0.578

**Table 6:** Mean PLCC, SROCC, and RMSE across 100 train–test combinations on the four considered databases using the logits or the features obtained from Extractor-Q and Extractor-S. In each column, the best and second-best values are marked in **boldface**.

	CVD2014			KoNViD-1k		
	PLCC ↑	SROCC ↑	RMSE ↓	PLCC ↑	SROCC ↑	RMSE ↓
Logits	0.856±0.037	0.843±0.038	11.333±1.138	0.773±0.021	0.763±0.023	0.413±0.016
Features (proposed)	0.859±0.036	0.844±0.039	11.129±1.214	0.786±0.021	0.779±0.024	0.401±0.017
	**LIVE-Qualcomm**			**LIVE-VQC**		
	**PLCC**↑	**SROCC**↑	**RMSE**↓	**PLCC**↑	**SROCC**↑	**RMSE**↓
Logits	0.742±0.075	0.705±0.082	8.203±0.831	0.755±0.040	0.729±0.046	11.250±0.634
Features (proposed)	0.755±0.071	0.721±0.077	8.037±0.803	0.775±0.037	0.744±0.044	10.854±0.578

## Data Availability

Not applicable.

## References

[1] Li Y., Meng S., Zhang X., Wang S., Wang Y., Ma S. UGC-VIDEO: Perceptual quality assessment of user-generated videos. Proceedings of the IEEE Conference on Multimedia Information Processing and Retrieval (MIPR).

[2] Yim J.G., Wang Y., Birkbeck N., Adsumilli B. Subjective quality assessment for youtube ugc dataset. Proceedings of the IEEE International Conference on Image Processing (ICIP).

[3] Khan Z.A., Beghdadi A., Cheikh F.A., Kaaniche M., Pelanis E., Palomar R., Fretland Å.A., Edwin B., Elle O.J. (2020). Towards a video quality assessment based framework for enhancement of laparoscopic videos. Medical Imaging 2020: Image Perception, Observer Performance, and Technology Assessment.

[4] Korhonen J. (2019). Two-Level Approach for No-Reference Consumer Video Quality Assessment. IEEE Trans. Image Process..

[5] Agarla M., Celona L., Schettini R. (2020). No-Reference Quality Assessment of In-Capture Distorted Videos. MDPI J. Imaging.

[6] Hosu V., Hahn F., Jenadeleh M., Lin H., Men H., Szirányi T., Li S., Saupe D. The Konstanz natural video database (KoNViD-1k). Proceedings of the International Conference on Quality of Multimedia Experience (QoMEX).

[7] Sinno Z., Bovik A.C. (2018). Large-scale study of perceptual video quality. IEEE Trans. Image Process..

[8] Ghadiyaram D., Pan J., Bovik A.C., Moorthy A.K., Panda P., Yang K.C. (2017). In-capture mobile video distortions: A study of subjective behavior and objective algorithms. IEEE Trans. Circuits Syst. Video Technol..

[9] Li D., Jiang T., Jiang M. Quality Assessment of In-the-Wild Videos. Proceedings of the ACM International Conference on Multimedia.

[10] He K., Zhang X., Ren S., Sun J. Deep residual learning for image recognition. Proceedings of the IEEE Conference on Computer Vision and Pattern Recognition (CVPR).

[11] Sandler M., Howard A., Zhu M., Zhmoginov A., Chen L.C. Mobilenetv2: Inverted residuals and linear bottlenecks. Proceedings of the IEEE Conference on Computer Vision and Pattern Recognition, Salt Lake City.

[12] Mittal A., Soundararajan R., Bovik A. (2013). Making a “Completely Blind” Image Quality Analyzer. IEEE Signal Process. Lett..

[13] Mittal A., Moorthy A.K., Bovik A.C. (2012). No-Reference Image Quality Assessment in the Spatial Domain. IEEE Trans. Image Process..

[14] Ghadiyaram D., Bovik A.C. (2017). Perceptual Quality Prediction on Authentically Distorted Images Using a Bag of Features Approach. Assoc. Res. Vis. Ophthalmol. J. Vis..

[15] Kundu D., Ghadiyaram D., Bovik A.C., Evans B.L. (2017). No-Reference Quality Assessment of Tone-Mapped HDR Pictures. IEEE Trans. Image Process..

[16] Saad M., Bovik A. Blind quality assessment of videos using a model of natural scene statistics and motion coherency. Proceedings of the Asilomar Conference on Signals, Systems and Computers (ASILOMAR).

[17] Xu J., Ye P., Liu Y., Doermann D. No-reference video quality assessment via feature learning. Proceedings of the International Conference on Image Processing (ICIP).

[18] Nuutinen M., Virtanen T., Vaahteranoksa M., Vuori T., Oittinen P., Häkkinen J. (2016). CVD2014—A database for evaluating no-reference video quality assessment algorithms. IEEE Trans. Image Process..

[19] Dendi S.V.R., Channappayya S.S. (2020). No-Reference Video Quality Assessment Using Natural Spatiotemporal Scene Statistics. IEEE Trans. Image Process..

[20] Ebenezer J.P., Shang Z., Wu Y., Wei H., Bovik A.C. No-Reference Video Quality Assessment Using Space-Time Chips. Proceedings of the IEEE International Workshop on Multimedia Signal Processing (MMSP).

[21] Korhonen J., Su Y., You J. Blind Natural Video Quality Prediction via Statistical Temporal Features and Deep Spatial Features. Proceedings of the ACM 28th ACM International Conference on Multimedia.

[22] Li Y., Po L., Cheung C., Xu X., Feng L., Yuan F., Cheung K. (2016). No-Reference Video Quality Assessment With 3D Shearlet Transform and Convolutional Neural Networks. IEEE Trans. Circuits Syst. Video Technol..

[23] Wang C., Su L., Zhang W. COME for No-Reference Video Quality Assessment. Proceedings of the IEEE Conference on Multimedia Information Processing and Retrieval (MIPR).

[24] Larson E.C., Chandler D.M. (2010). Most apparent distortion: Full-reference image quality assessment and the role of strategy. J. Electron. Imaging.

[25] Deng J., Dong W., Socher R., Li L.J., Li K., Fei-Fei L. Imagenet: A large-scale hierarchical image database. Proceedings of the IEEE Conference on Computer Vision and Pattern Recognition (CVPR).

[26] Cho K., van Merriënboer B., Gulcehre C., Bahdanau D., Bougares F., Schwenk H., Bengio Y. Learning Phrase Representations using RNN Encoder–Decoder for Statistical Machine Translation. Proceedings of the Conference on Empirical Methods in Natural Language Processing (EMNLP).

[27] Chen P., Li L., Ma L., Wu J., Shi G. RIRNet: Recurrent-In-Recurrent Network for Video Quality Assessment. Proceedings of the ACM International Conference on Multimedia.

[28] Li D., Jiang T., Jiang M. (2021). Unified Quality Assessment of in-the-Wild Videos with Mixed Datasets Training. Int. J. Comput. Vis..

[29] Tang Y., Jiang S., Xu S., Liu T., Li C. (2019). Blind Image Quality Assessment Based on Multi-Window Method and HSV Color Space. Appl. Sci..

[30] Ahmed Majeed H., Moaz H. A., Ramla Abdulnabi A. (2015). Quality Measurement of Blurred Images Using NMSE and SSIM Metrics in HSV and RGB Color Spaces. Phys. J..

[31] Gao X., Lu W., Tao D., Li X. (2010). Image quality assessment and human visual system. Visual Communications and Image Processing 2010.

[32] Bianco S., Celona L., Napoletano P., Schettini R. (2017). On the use of deep learning for blind image quality assessment. Signal Image Video Process..

[33] Siahaan E., Hanjalic A., Redi J.A. (2018). Semantic-aware blind image quality assessment. Signal Process. Image Commun..

[34] Ji W., Wu J., Shi G., Wan W., Xie X. (2019). Blind image quality assessment with semantic information. J. Vis. Commun. Image Represent..

[35] Bianco S., Cadene R., Celona L., Napoletano P. (2018). Benchmark Analysis of Representative Deep Neural Network Architectures. IEEE Access.

[36] Virtanen T., Nuutinen M., Vaahteranoksa M., Oittinen P., Häkkinen J. (2014). CID2013: A Database for Evaluating No-Reference Image Quality Assessment Algorithms. IEEE Trans. Image Process..

[37] Fang Y., Zhu H., Zeng Y., Ma K., Wang Z. Perceptual Quality Assessment of Smartphone Photography. Proceedings of the IEEE Conference on Computer Vision and Pattern Recognition (CVPR).

[38] Snyder D., Garcia-Romero D., Povey D., Khudanpur S. (2017). Deep Neural Network Embeddings for Text-Independent Speaker Verification.

[39] Paszke A., Gross S., Chintala S., Chanan G., Yang E., DeVito Z., Lin Z., Desmaison A., Antiga L., Lerer A. Automatic Differentiation in Pytorch. Proceedings of the 31st Conference on Neural Information Processing Systems (NIPS 2017).

[40] Pedregosa F., Varoquaux G., Gramfort A., Michel V., Thirion B., Grisel O., Blondel M., Prettenhofer P., Weiss R., Dubourg V. (2011). Scikit-learn: Machine Learning in Python. J. Mach. Learn. Res..

[41] He K., Zhang X., Ren S., Sun J. Delving deep into rectifiers: Surpassing human-level performance on imagenet classification. Proceedings of the IEEE International Conference on Computer Vision (ICCV).

[42] Shahriari B., Swersky K., Wang Z., Adams R.P., de Freitas N. (2016). Taking the Human Out of the Loop: A Review of Bayesian Optimization. Proc. IEEE.

[43] Thomee B., Shamma D.A., Friedland G., Elizalde B., Ni K., Poland D., Borth D., Li L.J. (2016). YFCC100M: The new data in multimedia research. Commun. ACM.

[44] Ye P., Kumar J., Kang L., Doermann D. Unsupervised feature learning framework for no-reference image quality assessment. Proceedings of the IEEE Conference on Computer Vision and Pattern Recognition.

